# State and Regional Trends in Incidence and Early Detection of Lung Cancer Among US Adults, 2010–2020

**DOI:** 10.5888/pcd21.240016

**Published:** 2024-07-25

**Authors:** Jonathan Bryant-Genevier, Christine M. Kava, Stephanie C. Melkonian, David A. Siegel

**Affiliations:** 1 Division of Cancer Prevention and Control, National Center for Chronic Disease Prevention and Health Promotion, Centers for Disease Control and Prevention, Atlanta, Georgia; 2 Epidemic Intelligence Service, Centers for Disease Control and Prevention, Atlanta, Georgia

**Figure:**
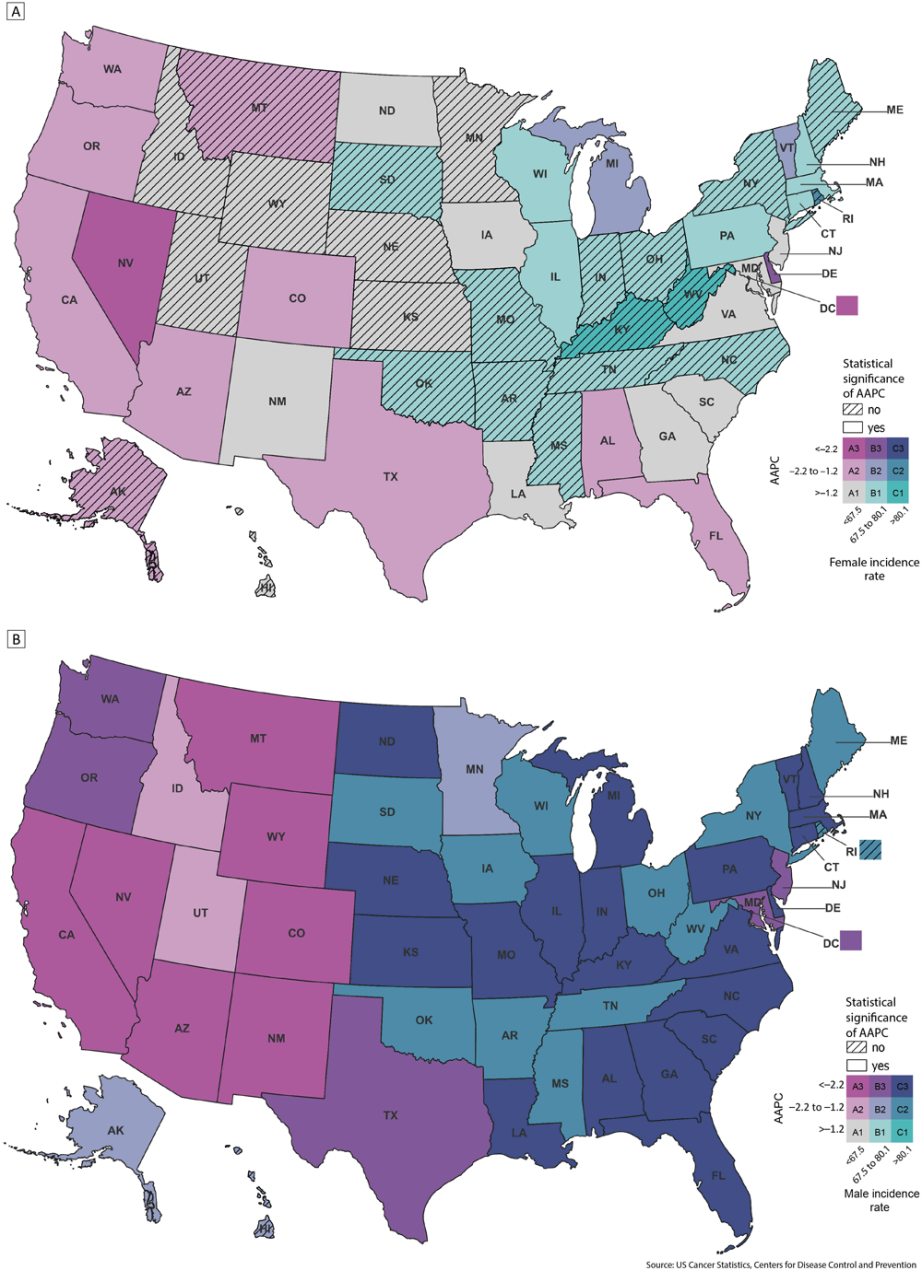
2010–2019 US age-adjusted state-level lung cancer incidence rates per 100,000 standard population with average annual percent change (AAPC) for female adults (Map A) and male adults (Map B). Incidence and AAPC range from lowest, A1, to highest, C3. Source: US Cancer Statistics, Centers for Disease Control and Prevention (10).

## Background

Lung cancer is the leading cause of cancer death in both male and female adults in the US (1,2). Overall incidence rates of lung cancer continue to decline following historical declines in cigarette smoking, estimated to account for roughly 90% of lung cancer cases (3,4). However, disparities in incidence persist among certain racial and ethnic groups and between sexes. For example, recent reports have shown rates remaining stable among female non-Hispanic Asian and Pacific Islander (NH-API) people and female non-Hispanic American Indian or Alaska Native (NH-AIAN) people (1).

Although recent advances in treatment, including targeted therapies, have improved mortality rates (5), early detection remains critical; survival is substantially higher among patients presenting with localized disease (4). Recent studies have shown that lung cancer screening, first recommended by the US Preventive Services Task Force (USPSTF) in 2013 for people at high risk for developing lung cancer, remains low (6) and suggest that geographic disparities exist in access to high-quality lung cancer screening facilities (7).

State and regional trends in lung cancer incidence and the proportion of cases diagnosed at localized-only stage have not been recently described (8,9). We measured geographic trends in incidence and the proportion of new cases diagnosed at localized-only stage, among male and female adults and by race and ethnicity, from 2010 through 2019.

## Data and Methods

We used data from the US Cancer Statistics (USCS) Incidence Analytic Database, which are from population-based registries that participate in the Centers for Disease Control and Prevention’s (CDC’s) National Program of Cancer Registries or the National Cancer Institute’s Surveillance, Epidemiology, and Results (SEER) Program and meet high-quality data criteria (10). Included registries covered approximately 100% of the US population from 2010 through 2019; data from 50 states and the District of Columbia were included (10). Annual incidence rates were calculated per 100,000 people and age-adjusted to the 2000 US standard population (19 age groups, Census P25–1130). Single year data from 2020 were analyzed separately, as the COVID-19 pandemic disrupted health services and may have contributed to declines in incidence; Indiana and Nevada were excluded from the 2020 analysis because the data for that year did not meet USCS standards of data quality. Cancer cases were staged using Merged Summary Stage categories; incidence rates and proportions of cases diagnosed at localized-only stage (ie, disease limited to the organ of origin) were calculated (10,11). Trends in rates and proportions from 2010 through 2019 were estimated by average annual percent change (AAPC) and 2-sided tests to determine if AAPCs had significant differences from zero; rates were described as increasing (AAPC > 0; *P* < .05), decreasing (AAPC < 0; *P* < .05), or stable (*P* > .05). Analyses were performed using SEER*Stat software (version 8.4.1, National Cancer Institute) and Joinpoint Regression Program (version 4.9.1.0, National Cancer Institute). To minimize racial misclassification of NH AI/AN populations, analyses among these populations used the USCS AI/AN Incidence Analytic Database and were restricted to purchased or referred care delivery area counties within or adjacent to federally recognized tribal lands (12,13).

## Highlights

Incidence rates declined 1.8% per year on average from 2010 through 2019, decreasing more rapidly among male adults (AAPC, −2.6%) than among female adults (AAPC, −1.0%). From 2010 through 2019, lung cancer incidence declined in 49 jurisdictions and remained stable in 2 jurisdictions among male adults and declined in 26 jurisdictions, remained stable in 23 jurisdictions, and increased in 2 jurisdictions among female adults. The lowest incidence and fastest declines in incidence were observed in the West (Table). From 2010 through 2019, rates among NH-API adults declined less than those among NH White, NH Black, and Hispanic adults across all geographic regions; in the Northeast, rates among NH API adults were stable from 2010 through 2019 (Table). The highest declines in incidence were observed among NH Black adults, although 10-year rates were higher than NH White adults in the Midwest and West.

**Table:** National and Regional Lung Cancer Incidence Rates and Average Annual Percentage Change, by Age, Sex, Race and Ethnicity, and Stage at Diagnosis, With Cases Diagnosed at Localized-Only Stage Stratified by Race and Ethnicity, US Cancer Statistics, 2010–2019 and 2020

Variable	US Department of Health and Human Services region														
	All			Northeast			Midwest			South			West		
	2020 Rate^a^^,^ b	10-Year rate^c^	AAPC^d^	2020 Rate	10-Year rate	AAPC	2020 Rate	10-Year rate	AAPC	2020 Rate	10-Year rate	AAPC	2020 Rate	10-Year rate	AAPC
**Overall**	57.9	72.8	−1.8	60.7	76.0	−1.4	67.1	80.9	−1.3	61.3	77.2	−1.9	41.7	54.9	−2.6
**Age, y**															
<40	1.1	1.3	−1.2	1.1	1.5	−0.9^e^	1.3	1.3	−2.3^e^	1.2	1.3	−0.8^e^	0.8	1.0	−0.8^e^
40−49	9.2	13.1	−5.3	10.2	14.3	−4.0	11.3	15.6	−5.2	9.9	14.6	−6.5	5.6	7.7	−3.7
50−59	50.3	67.3	−2.1	52.4	68.7	−1.9	61.9	77.8	−1.2	55.7	76.8	−2.5	29.1	40.3	−3.6
60−69	154.2	181.0	−1.7	159.1	186.9	−1.4	181.5	202.0	−0.9	168.4	198.2	−1.8	100.8	127.3	−2.8
70−79	261.6	335.2	−1.7	276.6	352.9	−1.4	296.1	371.7	−1.4	273.8	346.0	−1.5	197.5	266.0	−2.8
≥80	220.7	273.9	−1.1	231.4	287.0	−0.5^e^	245.9	288.7	−0.7	218.2	272.9	−1.2	192.9	247.8	−1.6
**Sex**															
Male	64.4	83.8	−2.6	65.7	84.9	−2.2	73.6	92.7	−2.2	70.4	92.3	−2.7	45.0	60.4	−3.3
Female	53.0	64.4	−1.0	57.3	69.8	−0.6	62.2	72.0	−0.4	54.2	65.4	−0.9	39.2	50.7	−1.9
**Race and ethnicity**															
NH White	63.2	78.2	−1.6	65.5	80.8	−1.2	69.2	82.7	−1.1	68.0	84.1	−1.6	45.9	60.2	−2.6
NH Black	58.6	76.5	−2.3	52.1	71.5	−2.7	72.6	92.2	−2.0	58.1	74.9	−2.1	50.0	67.1	−3.1
NH API	36.0	44.7	−1.0	39.1	48.9	0.3^e^	35.8	40.1	−1.3	29.8	37.7	−1.2	37.4	46.3	−1.3
NH AIAN^f^	53.8	68.7	−2.1	66.2	81.8	−1.3	73.7	88.0	−1.0	57.8	73.3	−1.7	43.8	58.0	−2.7
Hispanic	28.7	37.0	−1.8	36.0	46.4	−1.2	32.2	38.1	−2.2	29.3	37.1	−1.7	24.6	33.2	−2.1
**Stage at diagnosis^g^**															
Localized only	15.7	16.9	2.8	18.2	19.1	4.0	18.1	18.5	3.6	15.8	17.4	1.8	11.2	12.7	2.0
Regional	13.2	17.4	−2.2	13.4	18.1	−2.1	16.0	19.7	−1.5	14.2	18.8	−2.3	8.7	12.2	−3.2
Distant	27.1	36.1	−3.6	27.6	37.0	−3.6	31.4	40.8	−3.4	28.7	37.8	−3.3	20.3	27.7	−4.3
Unknown	2.0	2.5	−1.4	1.5	1.8	−2.2^e^	1.7	1.9	2.0	2.6	3.2	−2.0	1.5	2.3	−2.8^e^
**Localized-only stage at diagnosis, by race and ethnicity^h^**															
NH White	17.5	18.5	2.9	20.0	20.7	4.2	18.8	19.1	3.6	18.0	19.4	2.0	13.0	14.4	2.2
NH Black	14.0	15.5	2.3	13.2	15.3	2.6	17.5	19.0	2.9	13.7	14.3	2.4	11.4	13.7	1.8^e^
NH-API	8.0	9.2	3.8	10.8	12.0	5.3	8.0	7.9	2.1^e^	6.1	7.3	3.9^e^	7.7	9.0	3.0
NH-AIAN^f^	14.8	16.2	2.6	21.5	22.3	3.9	19.7	19.8	3.9	14.3	16.2	2.1	11.8	13.5	1.8
Hispanic	6.9	7.9	2.5	9.8	11.0	3.7	7.5	7.8	2.5^e^	6.6	7.8	2.4	5.8	6.7	1.8^e^

Abbreviations: AAPC, average annual percent change; AIAN, American Indian or Alaska Native; API, Asian or Pacific Islander; NH, non-Hispanic.

a Incidence rates calculated per 100,000 people, age-adjusted to the 2000 US standard population.

b 2020 Single-year incidence rates exclude data from Nevada and Indiana.

c 10-Year incidence rate uses data from 2010–2019.

d AAPC in incidence rate from 2010–2019, calculated using Joinpoint Regression Program; measures of trend (AAPC) were calculated using data years 2010–2019 only.

e Denotes calculated AAPCs that are not statistically different from 0% at significance level of *P* < .05.

f NH-AIAN populations were limited to individuals living in purchased or referred care delivery areas (12).

g Defined by Merged Summary Stage (11).

h Age-adjusted incidence rates of lung cancers diagnosed at localized-only stage, stratified by race and ethnicity.

In 2020, 25.5% of lung cancer cases among male adults and 30.6% among female adults, nationally, were diagnosed at localized-only stage. From 2010 through 2019, the proportion of cases diagnosed at localized-only stage rose among male (AAPC, 4.9%; 95% CI = 3.5%–6.2%) and female (AAPC, 4.5%; 95% CI = 3.3%–5.8%) adults (data not shown). State-level proportions of cases diagnosed at localized-only stage (Figure) were similar between male (range, 16.6%–24.7%) and female (range, 22.9%–31.0%) adults, with most jurisdictions showing similar increases across sexes from 2010 through 2019 (AAPC range: male adults, 2.6%–8.9%; female adults, 3.1%–7.6%). Overall, these data suggest a consistent trend toward earlier stage diagnoses among male and female adults; increases, however, varied by state.

**Figure:**
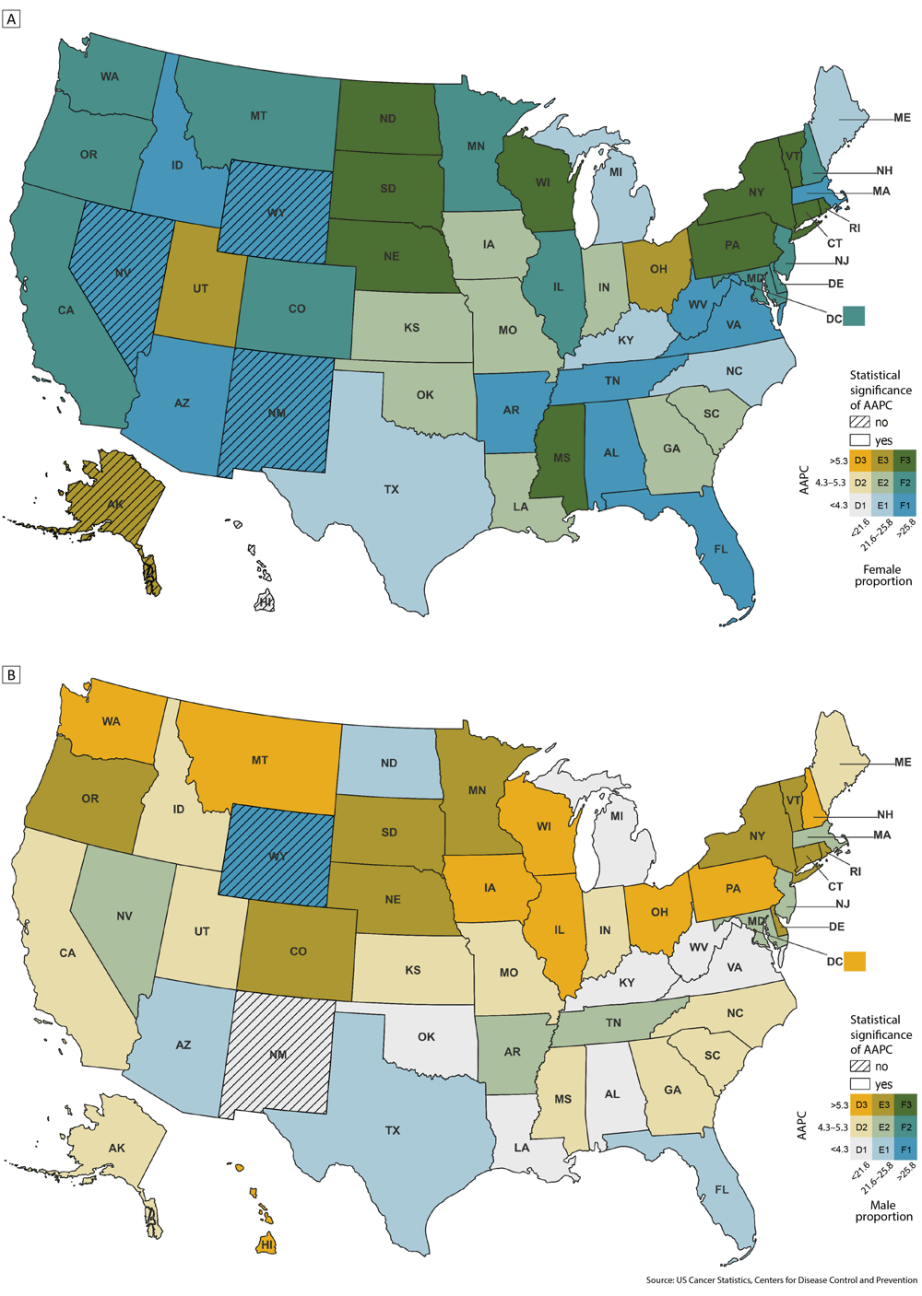
2010–2019 US state-level proportion of lung cancers diagnosed at localized-only stage with AAPC for female adults (Map A) and male adults (Map B). Proportions and AAPC range from lowest, D1, to highest, F3. Source: US Cancer Statistics, Centers for Disease Control and Prevention (10).

## Action

These maps describe recent state-specific trends and geographic variation in lung cancer incidence and early diagnosis in the US and present a benchmark for future work to evaluate implementation of USPSTF 2021 expanded lung cancer screening recommendations. Alongside contextual evidence, such as state and local tobacco control actions, these data may provide insight into future prevention strategies, facilitate programmatic development, and support tobacco control and lung cancer screening efforts (14). Increases in the proportion of lung cancer cases diagnosed at a localized-only stage coincide with the implementation of the 2013 USPSTF lung cancer screening recommendations and the US Centers for Medicare and Medicaid Services ensuring private insurance and Medicare coverage in 2015. Geographic variability in lung cancers diagnosed at localized-only stage may be attributable in part to differences in availability of screening facilities (7) or use of screening recommendations, specifically use of low-dose computed tomography, at the state level (6,15). These patterns differed from lung cancer incidence trends, which might reflect differences in population-based tobacco-control strategies, such as smoke-free laws and state tobacco control programs, which have been shown to reduce the prevalence of smoking.
